# Integrating Molecular Phenotyping into Treatment Algorithms for Advanced Oestrogen Receptor-Positive Breast Cancer

**DOI:** 10.3390/cancers17193174

**Published:** 2025-09-29

**Authors:** Sarah Childs, Ryoko Semba, Lucy Haggstrom, Elgene Lim

**Affiliations:** 1 The Kinghorn Cancer Centre, St Vincent’s Hospital Darlinghurst, Darlinghurst, NSW 2010, Australia; 2 Garvan Institute of Medical Research, Darlinghurst, NSW 2010, Australia; 3 Department of Breast Oncology, Juntendo University School of Medicine, Tokyo 113-8421, Japan; 4 School of Medicine and Health, University of New South Wales, Sydney, NSW 2033, Australia

**Keywords:** molecular testing, ctDNA, next-generation sequencing, precision medicine, endocrine resistance

## Abstract

Breast cancer is the most common malignancy and leading cause of cancer-related mortality among women worldwide. Oestrogen receptor (ER)-positive disease accounts for the majority of cases, where endocrine and targeted therapies have substantially improved survival. Nevertheless, resistance to therapy remains inevitable, emphasising the need for precision strategies informed by molecular profiling. The molecular landscape of ER-positive breast cancer is increasingly complex, characterised by diverse genomic alterations driving resistance and progression. Advances in next-generation sequencing and circulating tumour DNA (ctDNA) technologies enable the dynamic assessment of tumour heterogeneity and clonal evolution, informing prognostication and guiding biomarker-driven therapy. Uniquely, this review integrates molecular phenotyping with clinical treatment algorithms for advanced ER-positive breast cancer, providing a practical framework to translate genomic insights into patient care. Key genomic alterations and targeted strategies with demonstrated clinical benefit, including oral selective ER degraders (SERDs) and *PI3K/AKT/mTOR* inhibitors in selected biomarker populations, are highlighted. Emerging targets, such as human epidermal growth factor 2 (HER2) mutations, and the potential of ctDNA monitoring to detect resistance and guide therapeutic escalation are also discussed. Incorporating molecular profiling, as recommended by international guidelines, into routine clinical decision making can personalise therapy and optimise patient outcomes. Addressing real-world challenges, including cost and accessibility, will be critical to achieving equitable implementation of precision oncology for patients with ER-positive breast cancer worldwide.

## Simple Summary

Breast cancer is the most common cancer in women worldwide, and most cases are oestrogen receptor (ER) positive. These cancers are usually treated with hormone (endocrine) therapy and targeted drugs, which have greatly improved survival outcomes. However, many patients eventually stop responding to treatment, as the cancer develops resistance. Research has shown that ER-positive breast cancer is not one single disease but rather a group of subtypes driven by different genetic changes. New technologies, such as next-generation sequencing and blood tests that detect tumour DNA (ctDNA), allow for the identification of genetic differences. This can help guide more personalised treatment decisions. Promising new therapies include oral selective oestrogen receptor degraders and drugs targeting growth pathways such as PI3K/AKT/mTOR inhibitors. Wider access to molecular testing and ongoing drug development are essential to bring precision medicine to all patients.

## 1. Introduction

Globally, breast cancer is the most commonly diagnosed malignancy and the leading cause of cancer-related mortality among women [1,2]. Up to ~30% of high-risk early-stage patients develop metastatic disease, with risk persisting for decades; in ER-positive breast cancer, recurrences can occur up to twenty years following diagnosis, with nearly half occurring beyond five years post-endocrine therapy [3,4]. Survival rates vary widely among patients with a similar stage and subtype, complicating risk stratification and treatment decisions [1]. Expanding knowledge of the molecular heterogeneity of breast cancer has advanced precision oncology beyond conventional oestrogen receptor (ER), progesterone receptor (PR) and human epidermal growth factor 2 (HER2) subtyping toward therapies targeting tumour-specific molecular alterations to improve clinical outcomes [5]. This review integrates advancements in molecular phenotyping with practical treatment algorithms for advanced ER-positive breast cancer, providing a clinically focused framework that bridges emerging genomic insights with therapeutic decision making.

## 2. The Molecular Landscape of Advanced ER-Positive Breast Cancer

**Figure 1:**
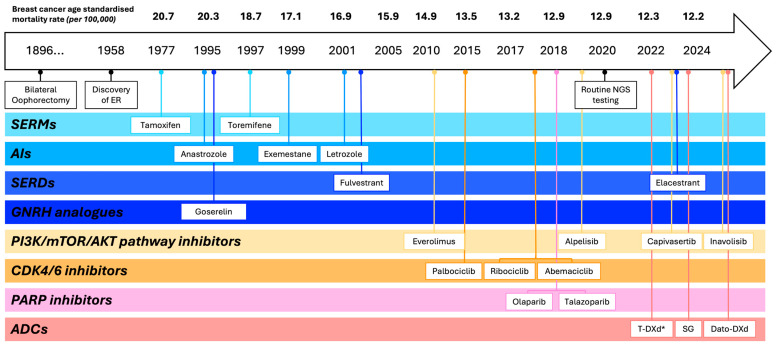
Timeline illustrating the evolution of FDA-approved endocrine and targeted therapies, categorised by mechanism of action or target, for advanced ER-positive breast cancer, alongside corresponding reductions in breast cancer-related mortality in Australia over time. Ref. [12] * Approved for HER2-low advanced breast cancer. Abbreviations: ADCs: antibody–drug conjugates; AIs: aromatase inhibitors; *AKT*: Protein kinase B; CDK4/6: cyclin-dependent kinase 4/6; Dato-DXd: Datopotamab deruxtecan; ER: oestrogen receptor; GNRH: gonadotropin-releasing hormone; *mTOR*: mechanistic target of rapamycin; NGS: next-generation sequencing; PARP: poly (ADP-ribose) polymerase; *PI3K:* phosphoinositide 3-kinase; SERDs: selective oestrogen receptor degraders; SERMs: selective oestrogen receptor modulators; SG: Sacituzumab Govitecan; T-DXd: Trastuzumab Deruxtecan.

Breast oncology was among the earliest fields to adopt targeted therapy, beginning with surgical oophorectomy [6,7] followed by the introduction of anti-oestrogenic agents for ER-positive disease and the identification of HER2 amplification as a therapeutic target [8]. Since then, the assessment of ER, PR and HER2 remains central for subtyping and guiding initial therapeutic decisions [9,10]. More recently, the incorporation of cyclin-dependent kinase 4/6 (CDK4/6) inhibitors to endocrine therapy has become a cornerstone treatment in both high-risk early-stage and advanced ER-positive breast cancer, significantly improving survival. The sequential evolution of hormone and targeted therapies for ER-positive breast cancer (Figure 1) has contributed to a sustained decline in breast cancer-related mortality [11,12]. However, resistance remains inevitable, highlighting the need for novel treatment strategies. Trials such as MAINTAIN and postMONARCH have evaluated the continuation or rechallenge of CDK4/6 inhibitors in selected patients, showing modest improvements in progression-free survival (PFS), particularly after a prolonged response to CDK4/6 inhibition [13,14].

**Figure 2:**
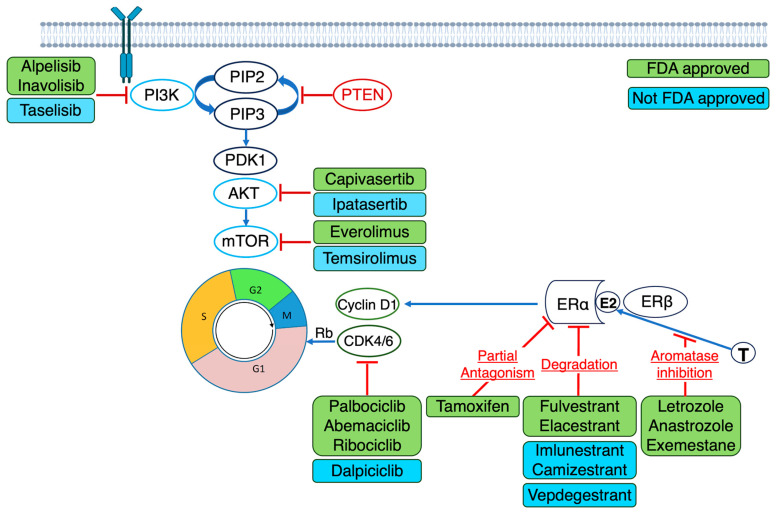
Key oncogenic signalling pathways implicated in advanced ER-positive breast cancer and corresponding targeted therapeutic strategies with positive phase III trial data. Evidence for PIK3CA inhibitors emerged from the SOLAR-1 [18] and BYLieve [19] trials, where alpelisib in the second-line setting improved PFS in PIK3CA-mutant patients (8–11 months; HR 0.65, *p* < 0.001). Mutant-selective PIK3CA inhibitors, such as inavolisib, reduce off-target toxicities and demonstrated improved PFS (15 vs. 7.3 months; HR 0.43, 95% CI 0.32–0.59 and *p* < 0.001) and OS (34.0 vs. 27.0 months; HR 0.67, *p* = 0.019) in the first-line setting (INAVO120) [20]. Capivasertib, a pan-AKT inhibitor, showed efficacy in the second-line setting, irrespective of AKT pathway alterations (PFS 7.2 vs. 3.6 months; HR 0.60, 95% CI 0.51–0.71 and *p* < 0.001) [21]. Everolimus combined with exemestane improved PFS in the second-line setting in the BOLERO-2 trial (HR 0.36, 95% CI 0.27–0.47 and *p* < 0.001) [22], prior to the routine implementation of molecular profiling. CDK4/6 inhibitors in combination with endocrine therapy remain the cornerstone of first-line treatment, irrespective of molecular mutation status. Abbreviations: *AKT*: Protein kinase B; CDK4/6: cyclin-dependent kinase 4/6; E2: oestradiol; ERα: oestrogen receptor alpha; ERβ: oestrogen receptor beta; FDA: Food and Drug Administration; *mTOR*: mammalian target of rapamycin; PDK1: 3-phosphoinositide-dependant kinase 1; PI3K: phosphoinositide 3-kinase; PIP2: Phosphatidylinositol (4,5)-bisphosphonate; PIP3: Phosphatidylinositol (3,4,5)-Triphosphonate; *PTEN*: Phosphatase and tensin homolog; Rb: Retinoblastoma; T: testosterone.

Advances in molecular profiling have revealed key truncal and acquired alterations in ER-positive breast cancer. Frequently observed alterations include mutations in *ESR1* (10–40%), *TP53* (10–34%), *PIK3CA* (30%), *CCND1* amplifications (9–22%), *GATA3* (12–20%), *MAP3K1* (8–10%), *FGFR1* amplifications (10–15%), *CDH1* (9%), *PTEN* (7%)*, AKT1* (7%), germline *BRCA1/2* (4%) and germline *PALB2* (1%) [15,16,17]. These mutations drive diverse biological processes including endocrine resistance, *PI3K/AKT/mTOR* signalling, cell cycle regulation and chromatin remodelling, influencing both prognosis and therapeutic response [15,16,17]. Alterations such as *PIK3CA* and *ESR1* are actionable, with approved or emerging therapies, while others such as *TP53* and *GATA3* are primarily prognostic or mechanistic [15,16,17]. Figure 2 outlines key signalling pathways and corresponding targeted therapies supported by phase III trial data.

International guidelines recommend comprehensive genomic profiling, either tumour or circulating tumour DNA (ctDNA), in advanced ER-positive breast cancer [23,24,25]. The American Society of Clinical Oncology (ASCO) supports utility beyond the first-line setting to influence therapeutic decision making [23]. In 2020, the European Society for Medical Oncology (ESMO) updated guidelines to recommend routine testing when results are likely to influence treatment selection, guided by the ESMO Scale for Clinical Actionability of Molecular Targets (ESCAT) framework, which stratifies molecular alterations by strength of clinical evidence [24,26]. Table 1 summarises clinically relevant alterations in ER-positive breast cancer according to ESCAT tiers [24,26].

Mutations in the ligand-binding domain of the *ESR1* gene, which encodes ERα, represent a well-established mechanism of acquired endocrine resistance [36]. These mutations result in constitutive ERα activation, driving oestrogen-independent growth [10,36]. The most common *ESR1^mut^*, along with their prevalence and clinical implications, are summarised in Table 2. Their frequency increases with longer exposure to endocrine therapy and is associated with poorer results, with outcomes varying depending on the specific *ESR1^mut^* and the presence of dual mutations [36,37,38]. The most prevalent *ESR1^mut^* include D538G and Y537S; other variants include Y537N, Y537C, L536H, L536P, L536R, S463P and E380Q [36].

The *PIK3CA* gene has emerged as a clinically relevant biomarker in advanced ER-positive breast cancer [18,40]. Activating mutations in *PIK3CA* result in hyperactivation of the *PI3K/AKT/mTOR* pathway, promoting oestrogen-independent growth [10,40]. These mutations are among the most common in advanced ER-positive breast cancer, occurring in 28–46% of cases, and are typically truncal, present in the primary tumour at diagnosis [40,41]. Discordance of *PIK3CA^mut^* between the primary tumour and metastatic sites is infrequent at 9.8% (95% CI, 7–13%) [41]. *PIK3CA^mut^* are associated with poor prognosis (HR 1.2, 95% CI 0.9–1.5 and *p* < 0.001), with a meta-analysis demonstrating an 8-month OS difference, and chemotherapy resistance [10,40,42]. The most frequent *PIK3CA^mut^* include H1047R (35%), E545K (17%) and E542K (10%), with biochemical differences, though current evidence does not link them to differential clinical outcomes [18,43]. A subset of tumours harbour multiple *PIK3CA^mut^*, possibly reflecting clonal evolution and greater pathway activation, though prognostic implications remain uncertain [43]. Co-occurring *PIK3CA^mut^* and *ESR1^mut^*, observed in 10–15% of endocrine-resistant cases, may confer synergistic resistance [44,45]. Other *PI3K* pathway alterations, including *AKT1^mut^* and *PTEN^del^*, each with an estimated incidence of 6%, are implicated in endocrine resistance and represent emerging therapeutic targets [5,10,26].

Additional clinically actionable alterations include *BRCA1/2^mut^*, which are present in ~4–5% of ER-positive breast cancers, the majority of which (~75%) are germline [10]. *BRCA1/2^mut^* tumours exhibit defective homologous recombination repair and are sensitive to poly-(ADP-ribose)-polymerase (PARP) inhibitors, offering a therapeutic option in early and advanced disease. Although associated with genomic instability and aggressive biology, the prognostic impact of *BRCA1/2^mut^* varies across studies, and sensitivity to PARP inhibitors may provide an important counterbalance to their adverse biological features [30,31]. Similarly, germline *PALB2^mut^,* occurring in 1–2% of ER-positive breast cancers, impair homologous recombination repair and confer comparable sensitivity to PARP inhibitors [26].

**Figure 3:**
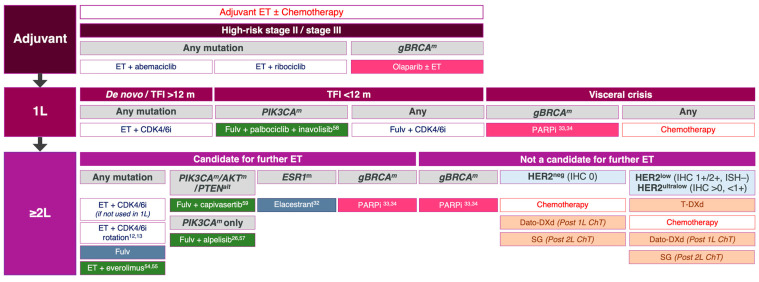
Treatment algorithm for ER-positive, HER2-negative advanced breast cancer integrating molecular profiling and targeted therapies, based on pivotal phase II and III clinical trial data. Abbreviations: *AKT*: Protein kinase B; CDK4/6i: cyclin-dependant kinase 4/6 inhibitor; ChT: chemotherapy; Dato-DXd: Datopotamab deruxtecan; *ESR1:* oestrogen receptor 1; ET: endocrine therapy; Fulv: fulvestrant; *gBRCA:* germline breast cancer gene; HER2: human epidermal growth factor 2; IHC: immunohistochemistry; PARPi: poly (ADP-ribose) polymerase inhibitors; *PIK3CA:* phosphatidylinositol-4,5-bisphosphonate 3-kinase catalytic subunit alpha; *PTEN*: Phosphatase and tensin homolog; SG: Sacituzumab Govitecan; T-DXd: Trastuzumab Deruxtecan; TFI: treatment-free interval.

Somatic *HER2^mut^*, distinct from *HER2* amplification, occur in 3–6% of ER-positive ductal and 18–26% of pleomorphic lobular carcinomas [10,46]. These mutations promote endocrine resistance through ER-HER2 crosstalk and are associated with shorter PFS and reduced endocrine sensitivity [10,47]. With the emergence of molecular targets, the contemporary management of advanced ER-positive breast cancer (Figure 3) has shifted to prioritise biomarker-directed therapies over chemotherapy or antibody–drug conjugates (ADCs) when actionable targets are identified.

## 3. Molecular Profiling in Advanced ER-Positive Breast Cancer

Molecular profiling plays a central role in advanced ER-positive breast cancer, supporting prognostication, real-time monitoring of therapeutic response and identification of actionable alterations for targeted therapy [10,48]. It can be performed using tumour tissue or ctDNA from blood, referred to as a *liquid biopsy* [8]. While tissue biopsy remains the gold standard for initial diagnosis and immunohistochemical (IHC) profiling of ER, PR and HER2, it may inadequately capture spatial and temporal tumoural heterogeneity [10]. Distinct metastases can harbour different mutations, and resistance alterations can emerge under therapeutic pressure [48,49]. Up to 40% of tumours switch molecular subtype upon progression, which has prompted guideline recommendations to consider repeat biopsy where feasible [10,25]. Subtype switching with loss of ER expression occurs in 10–20% of cases, whilst HER2 status changes in 5–15%, most frequently as HER2 gain [50,51,52]. Such changes are clinically relevant as they may confer resistance to endocrine therapy, alter prognosis or open eligibility for targeted agents, such as HER2-directed therapies in cases of acquired HER2 expression. However, repeat tissue sampling is not always feasible, as is limited by procedural risks, anatomical inaccessibility and patient quality-of-life implications [10,48].

**Figure 4:**
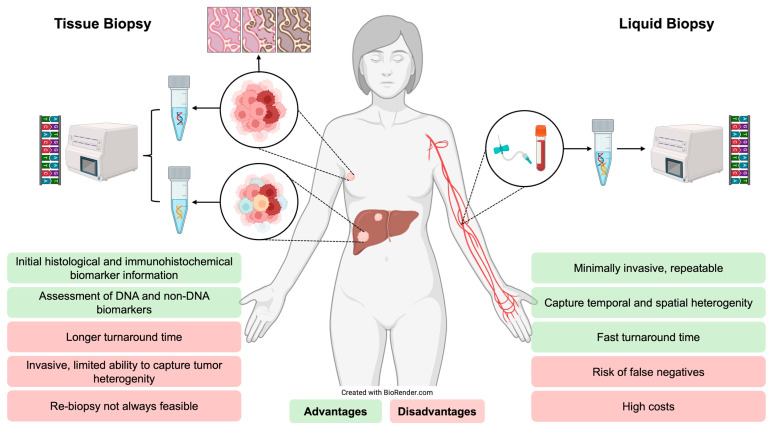
Advantages and disadvantages of tumour tissue biopsy compared with liquid biopsy for molecular profiling in advanced ER-positive breast cancer. This figure compares traditional tissue biopsy with liquid biopsy (ctDNA) approaches. Tissue biopsy provides histopathological context and comprehensive genomic information but is invasive, may require repeat procedures and can fail to represent tumour heterogeneity or clonal evolution. Liquid biopsy offers a minimally invasive alternative, which offers real-time assessment of tumour dynamics, enabling the detection of resistance mutations or monitoring of treatment response. Sensitivity of ctDNA can be lower, particularly in low-volume or early-stage disease.

In this context, plasma-based ctDNA profiling offers a minimally invasive alternative that captures tumour heterogeneity and molecular evolution [48]. Cell-free DNA (cfDNA) is mainly released via apoptosis and necrosis, a proportion of which is derived from tumour cells (ctDNA) [10,49]. The ctDNA fraction varies from 0.01 to 0.1% in early-stage disease to 5 to 10% in advanced disease, influenced by tumour burden, proliferative rate and breast cancer subtype [10,49]. Detection methods include digital droplet polymerase chain reaction (ddPCR) for precise detection of known mutations or next-generation sequencing (NGS) for broad multi-gene profiling [10,37,49]. Liquid biopsy enables repeat, relatively non-invasive and real-time assessment and quantification of genomic alterations to capture intra-tumoural heterogeneity and clonal evolution, although it can be limited by false-negative results from low-shedding tumours [10,48,53]. Figure 4 summarises the advantages and limitations of tissue versus liquid biopsy for molecular profiling in ER-positive breast cancer.

Retrospective studies report ~60% concordance between tissue- and plasma-based molecular profiling, with ~20% of variants unique to either source [48]. *ESR1^mut^* are the most frequent mutations exclusive to ctDNA (55%), while *PIK3CA^mut^* demonstrated the highest concordance (70%) [48]. The prospective plasmaMATCH trial reported 93% sensitivity for ctDNA detection of *ESR1*, *PIK3CA*, *HER2* and *AKT1* mutations compared to tissue sequencing, which increased to 98% with contemporaneous sampling [54]. Meta-analyses confirm high sensitivity and specificity for ctDNA detection of *ESR1^mut^* (sensitivity 75.5%; specificity 88.2%) and *PIK3CA^mut^* (sensitivity 73%; specificity 83%) [55,56]. Dynamic ctDNA monitoring has been proposed as a surrogate biomarker of treatment efficacy. In MONALEESA-3, ctDNA changes between cycles 1 and 4 correlated strongly with PFS (HR 0.29, 95% CI 0.22–0.38 and *p* < 0.0001) and OS (HR 0.23, 95% CI 0.17–0.31 and *p* < 0.0001) [57].

Together, these findings support ctDNA as a valuable tool for molecular profiling in advanced ER-positive breast cancer, although tissue biopsy remains critical for initial diagnosis and IHC assessment [54].

## 4. Therapies Targeting Genomic Aberrations in Advanced ER-Positive Breast Cancer

Genomic profiling plays a critical role in identifying targetable alterations in advanced ER-positive breast cancer, offering the potential to improve clinical outcomes [48]. Several mutations are classified as ESMO ESCAT tier I or II, denoting readiness for clinical use or promising investigational therapies (Table 1) [26]. The identification of tumour-specific oncogenic driver mutations has triggered a surge in drug development and reshaped the clinical trial landscape (Figure 3).

### 4.1. ESR1 Mutations

New therapies that degrade ERs have been developed to retain clinical activity in *ESR1^mut^* by targeting both mutant and wild-type ERα, unlike conventional endocrine therapies such as AIs [10,58]. The phase III SoFEA and EFECT trials demonstrated that *ESR1^mut^* predicted poor response to AIs, with improved outcomes using fulvestrant, a first-generation intramuscular selective ER degrader (SERD) (median PFS 2.4 vs. 3.9 months; HR 0.59, *p* = 0.01) [58]. However, the efficacy of fulvestrant has been limited by poor bioavailability and limited dosing [10,46].

Elacestrant, a next-generation oral SERD, received FDA approval based on the phase III EMERALD trial [9,10,28]. Elacestrant significantly improved PFS compared to standard endocrine monotherapy in patients who progressed with endocrine therapy and a CDK4/6 inhibitor (HR 0.70, 95% CI 0.55–0.88 and *p* = 0.002), with greater benefit observed in *ESR1^mut^* tumours (HR 0.55, 95% CI 0.39–0.77 and *p* = 0.0005) [28]. In a post hoc analysis, a longer duration of prior CDK4/6 inhibitor (>12 months) was predicted for superior PFS of 8.6 months in those receiving elacestrant vs. 1.9 months for those receiving standard endocrine therapy (HR 0.41, 95% CI 0.26–0.63 and *p* = 0.014) [59]. These results informed FDA approval criteria and highlight the importance of integrating molecular characteristics and functional response to prior therapy to optimise patient selection for second-line endocrine monotherapy.

The phase II SERENA-2 trial evaluated camizestrant, an oral SERD, in the second-line setting, demonstrating superior PFS compared with fulvestrant in the overall population (7.2 vs. 3.7 months; HR 0.59, 90% CI 0.42–0.82 and *p* = 0.0170) [60]. This correlated with early reductions of ctDNA *ESR1* variant allele frequency (VAF) by cycle 2 [60]. Other oral SERDs investigated in the second-line setting include imlunestrant (phase III EMBER-3 trial) and giredestrant (phase II acelERA trial), both demonstrating benefits limited to the *ESR1^mut^* population [61,62].

In the phase III VERITAC trial, vepdegestrant, an ER proteolytic-targeting chimera (PROTAC) degrader, demonstrated superior efficacy to fulvestrant in the *ESR1^mut^* population (PFS 5.0 vs. 2.1 months; HR 0.58, *p* < 0.001) [63].

Given the benefit of oral SERDs in advanced disease, several phase III trials (LiDERA (NCT04961996), EMBER-4 (NCT05514054) and ELEGANT (NCT06492616)) are investigating switching from AIs to SERDs in high-risk early breast cancer. Table 3 summarises completed and ongoing phase II and III trials investigating novel endocrine therapies stratified by *ESR1^mut^* status.

Serial ctDNA monitoring has detected emerging *ESR1^mut^* at a median of 6.7 months before radiographic progression [46]. The phase III PADA-1 and SERENA-6 trials enrolled patients on first-line AI plus CDK4/6 inhibitor therapy with rising *ESR1^mut^* on ctDNA (by NGS) without radiographic progression [29,68]. Patients were randomised to continue their current therapy or switch the endocrine therapy backbone to fulvestrant (PADA-1) or camizestrant (SERENA-6) while continuing their CDK4/6 inhibitor [29,68]. Both trials demonstrated improved PFS with early switching (PADA-1: 11.9 vs. 5.7 months, HR 0.61, 95% CI 0.43–0.86 and *p* = 0.004; SERENA-6: 16.0 vs. 9.2 months; HR 0.44, 95% CI 0.31–0.60 and *p* < 0.00001) [29,68]. Patients were monitored with ctDNA testing every 2–3 months; however, only 10–17% of patients were randomised to escalation of therapy, making this approach resource intensive and cost prohibitive for routine implementation into clinical practice [29,68].

Ongoing trials such as persevERA and SERENA-4 are evaluating whether upfront SERDs can prevent *ESR1^mut^*-driven resistance, which, if positive, may represent an alternative strategy, avoiding the need for serial ctDNA testing [69,70].

### 4.2. Alterations in PIK3CA, AKT and PTEN

The *PI3K/AKT* signalling pathway plays a critical role in various physiological processes, including cell growth, proliferation, survival and the regulation of glucose and lipid metabolism [72]. Consequently, therapies targeting this pathway have a narrow therapeutic index, and tolerability has proven clinically challenging.

Everolimus, an mTOR inhibitor, was the first *PI3K/AKT* pathway-directed therapy approved in breast cancer. The phase III BOLERO-2 trial demonstrated a PFS benefit of 10.6 months with exemestane plus everolimus vs. 4.1 months with exemestane alone (HR 0.36, 95% CI 0.27–0.47, *p* < 0.001) in patients who had progressed on endocrine therapy [38]. The phase II PrE0102 study similarly demonstrated a PFS benefit of second-line fulvestrant plus everolimus of 10.3 months vs. 5.1 months with fulvestrant monotherapy (HR 0.61, 95% CI 0.40–0.92 and *p* = 0.02) [73]. Both trials predated routine NGS profiling and CDK4/6 inhibitor use, therefore limiting applicability in the current clinical landscape. A recent single-arm study evaluating fulvestrant plus everolimus post-CDK4/6 progression demonstrated a median PFS of 6.8 months and validated ctDNA dynamics as a prognostic biomarker [74].

Alpelisib, an oral α-selective *PIK3CA* inhibitor, demonstrated efficacy in the phase III SOLAR-1 trial when combined with fulvestrant versus fulvestrant monotherapy in the second-line setting for advanced ER-positive/HER2-negative breast cancer [18]. This combination achieved a 45% reduction in the risk of progression and a 7.9-month improvement in OS, although it did not reach the pre-specified threshold for statistical significance [18]. However, 90% of patients in SOLAR-1 had not received a CDK4/6 inhibitor prior, which is now standard first-line therapy [18]. The phase II BYLieve trial, assessing alpelisib post-progression on a CDK4/6 inhibitor, demonstrated a median PFS of 7.5 months and an OS ranging from 20.7 to 29.0 months [19]. Based on these results, alpelisib received FDA approval for patients with advanced *PIK3CA^mut^* ER-positive/HER2-negative breast cancer. However, high toxicity rates including grade ≥ 3 hyperglycaemia (36%), grade ≥ 3 rash (10%), 25% discontinuation rate and 64% dose reductions and/or interruptions have limited clinical utility [18,19]. Hyperglycaemia is the most frequent adverse event, impacting up to 60% of patients receiving alpelisib, and safety in patients with type 1 or 2 diabetes has not been established. Use of prophylactic metformin has been shown to reduce the incidence and severity of alpelisib-induced hyperglycaemia, any grade (44.1%) and grades 3–4 (5.9%), in the METALLICA study [75], facilitating continuation of therapy.

Next-generation mutant selective *PI3Kα* degraders, such as inavolisib, aim to spare wild-type *PI3K* signalling, thereby reducing off-target toxicities [20]. The phase III INAVO120 trial evaluated inavolisib or placebo with fulvestrant and palbociclib in the first-line metastatic *PIK3CA^mut^* patients who relapsed on or within 12 months of adjuvant endocrine therapy [20]. Inavolisib significantly improved PFS (15 vs. 7.3 months; HR 0.43, 95% CI 0.32–0.59 and *p* < 0.001) and OS (34.0 vs. 27.0 months; HR 0.67, *p* = 0.019), marking the first *PIK3CA* pathway therapy to demonstrate an OS benefit in breast cancer and underscoring its potential to change first-line treatment standards [20]. Inavolisib was also more tolerable when compared to alpelisib, with grade ≥ 3 hyperglycaemia (6%), grade ≥ 3 rash (2.5%) and a 7% discontinuation rate [20]. This study has shifted the mutation testing paradigm to *before* first-line metastatic therapy for some patients. Early-phase trials investigating novel, mutant-specific *PIK3CA* inhibitors such as RLY-2608 (NCT05216432) and STX-478 (NCT05768139) have demonstrated promising efficacy and tolerability and are now progressing into phase III evaluation.

The phase III CAPItello-291 trial evaluated capivasertib, a pan-AKT inhibitor, plus fulvestrant vs. fulvestrant monotherapy in the second-line setting for advanced ER-positive/HER2-negative breast cancer, regardless of but stratified by mutational status, with 41% of patients harbouring an *AKT* pathway alteration (defined as *PIK3CA^mut^*, *AKT^mut^* and/or *PTEN^del^*) [21]. Capivasertib significantly prolonged PFS in the intention-to-treat (ITT) population (7.2 vs. 3.6 months; HR 0.60, 95% CI 0.51–0.71 and *p* < 0.001) and the pathway-altered population (7.2 vs. 3.6 months; HR 0.50, 95% CI 0.32–0.59 and *p* < 0.001) [21]. Despite similar efficacy, FDA approval was granted *only* for patients with an identified pathway alteration. Capivasertib was relatively well tolerated with grade ≥ 3 hyperglycaemia (2%), grade ≥ 3 rash (12%) and a 13% discontinuation rate [21]. A phase III study of capivasertib plus fulvestrant and a CDK4/6 inhibitor in the first-line metastatic setting is currently underway (NCT04862663). The phase III FINER trial assessed ipatasertib, an alternative pan-AKT inhibitor, plus fulvestrant in the second-line setting, stratified by *AKT* pathway alterations [76]. Ipatasertib improved PFS in the ITT population (5.3 vs. 1.9 months; HR 0.61, 95% CI 0.46–0.81 and *p* = 0.0007), with greater benefit in the mutant cohort (5.5 vs. 1.9 months; HR 0.47, 95% CI 0.31–0.72 and *p* = 0.0005) [76].

Table 4 summarises completed and ongoing phase II and III trials investigating therapies targeting the *PIK3CA/PTEN/AKT* pathway in advanced breast cancer.

### 4.3. BRCA1/2 and PALB2 Mutations

The benefit of PARP inhibitors for patients with germline *BRCA1/2^mut^* is well established, supported by phase III trials OlympiAD and EMBRACA [30,31]. OlympiAD demonstrated improved PFS with olaparib vs. chemotherapy (7.0 vs. 4.2 months; HR 0.58, *p* < 0.001) in advanced HER2-negative breast cancer (~50% ER positive) [30]. EMBRACA demonstrated superior PFS with talazoparib (PFS 8.6 vs. 5.6 months; HR 0.54, *p* < 0.001) [31]. While the efficacy of PARP inhibitors in somatic *BRCA1/2^mut^* remains under investigation, a phase II olaparib trial suggested comparable activity, with an overall response rate (ORR) of 50% [32]. This trial also demonstrated benefit from olaparib in patients with a germline *PALB2^mut^* (ORR 82%) [32]. Ongoing studies are investigating PARP inhibitors in earlier treatment settings and novel combinations, including with oral SERDs, as in the EvoPAR-Breast01 trial (NCT06380751).

### 4.4. HER2 Mutations

Trials specific to *HER2^mut^* breast tumours are limited due to their rarity, with most evidence arising from pan-tumour basket trials. While FDA-approved therapies exist for ER-positive/HER2-non-amplified breast cancer based on HER2 IHC expression, no FDA-approved therapies currently cover *HER2^mut^* breast cancers specifically. Furthermore, in practice, *HER2^mut^* testing is not yet routine, as it requires NGS panels rather than standard IHC or in situ hybridisation (ISH) testing.

The phase II SUMMIT trial evaluated neratinib, a pan-HER tyrosine kinase (TKI), as monotherapy or combined with fulvestrant—with or without trastuzumab—in patients with advanced *HER2^mut^* ER+/HER2-negative breast cancer who had progressed on prior CDK4/6 inhibitor [33]. The triplet regimen achieved an ORR of 39% and a median PFS of 8.3 months [33]. Trastuzumab deruxtecan (T-DXd), an ADC consisting of a HER-2 targeted monoclonal antibody combined via a cleavable peptide linker to a topoisomerase I inhibitor payload, has demonstrated durable responses in heavily pre-treated *HER2^mut^* non-small-cell lung cancer (NSCLC) in the DESTINY-Lung01 trial, leading to FDA approval [79]. In the DESTINY-Pantumour01 basket trial, T-DXd demonstrated a promising ORR of 50% in the *HER2^mut^* breast cancer cohort [34]. A phase I trial (NCT05372614) is currently evaluating the combination of T-DXd with Neratinib in patients with HER2 amplification or *HER2^mut^* [80].

## 5. Current Limitations and Future Directions of a Molecular Phenotypic Approach to Treating ER-Positive Breast Cancer

Despite growing clinical evidence and support from international guidelines, several key limitations hinder the widespread integration of molecular profiling in advanced ER-positive breast cancer. High upfront costs of NGS panels, limited reimbursement pathways and variation in test availability restrict patient access. Furthermore, access to matched targeted therapies remains largely confined to clinical trials or cost-share programs, further limiting accessibility, particularly in under-resourced settings.

Implementing molecular profiling requires specialised laboratory infrastructure, bioinformatics support and clinical expertise to interpret results. Tissue-based NGS is more established but often involves delays, limiting utility in time-sensitive scenarios, and may require repeat biopsies, which are not always feasible or acceptable. ctDNA offers a minimally invasive, real-time alternative, though sensitivity is limited in early-stage, low-volume or ER-positive subtypes. ddPCR is a cost-effective, highly sensitive method for detecting known mutations, but it has not been widely used in pivotal phase III clinical trials; thus, comparative studies would be required to facilitate widespread expansion of this technology. Effective molecular profiling requires a nuance understanding of the technical limitations and the complex, non-binary implications of genomic data to provide personalised treatment recommendations.

To address these challenges, health systems are increasingly adopting centralised testing models, multidisciplinary molecular tumour boards and partnerships with reference laboratories to standardise reporting and facilitate expert input. Centralised testing can achieve economies of scale and decrease per-sample costs, while clinical trial participation may provide subsidised or no-cost testing opportunities. Ultimately, demonstrating cost effectiveness of molecular profiling will be essential to support broader government or insurance reimbursement. A pragmatic strategy to improve the cost effectiveness in ER-positive breast cancer is to prioritise a focused panel testing approach limited to the most common, currently actionable molecular targets, such as *ESR1*, *PIK3CA*, *AKT*, *PTEN*, *BRCA1/2* and *HER2*, acknowledging that a small percentage of variants may be missed with this methodology. Investment in clinician education and local expertise through collaborative partnerships with academic centres will build capacity and ensure the integration of molecular profiling into routine practice.

The use of molecular profiling in clinical care raises important ethical considerations. Informed consent processes must clearly explain the scope of testing, potential incidental findings and implications for family members. Robust safeguards for data privacy and cybersecurity are essential, as genomic information constitutes highly sensitive personal data. Clear policies on data sharing, storage and secondary use are necessary to protect patient autonomy while supporting research and innovation. Addressing these ethical challenges proactively will help ensure that precision medicine advances in a way that maintains public trust and protects patient rights.

Future molecular profiling in advanced ER-positive breast cancer aims to improve therapeutic precision by expanding actionable alterations. Beyond guiding targeted therapies, it may define endocrine therapy duration, identify late recurrence risk, personalise surveillance and adapt treatment to resistance mutations. Early evidence supports therapeutic escalation with the emergence of detectable *ESR1^mut^*, and future studies should explore expanding this approach to other mutational alterations that may emerge with therapy selection. For example, *HER2^mut^* detection may warrant early change to HER2-directed TKIs or ADCs; somatic *BRCA1/2^mut^* could support early introduction of PARP inhibitors; Retinoblastoma (Rb) loss, implicated in CDK4/6 inhibitor resistance, may justify earlier second-line therapy; and cyclin E amplification may predict benefit from early CDK2 inhibitors. Emerging CDK2 inhibitors are being developed to address resistance to CDK4/6 inhibition, particularly in the setting of cyclin E amplification or Rb loss. Several selective CDK2 inhibitors, such as PF-07104091 (NCT04553133) [81] and BLU-222 (NCT05252416) [82], are currently in phase I/II clinical trials in the second line and beyond. Early results have demonstrated acceptable tolerability and preliminary anti-tumour activity, with a disease control rate of 61.5% in one cohort [81], supporting ongoing development.

Looking ahead, integrating ctDNA kinetics with clinical features and artificial intelligence driven analytics could enhance real-time treatment decisions, enabling therapy escalation or de-escalation based on tumour evolution and early detection of resistance. These dynamic risk models have the potential to refine risk stratification, optimise sequencing of targeted therapies and improve clinical outcomes for patients with advanced ER-positive breast cancer. However, prospective validation is required to confirm their clinical utility and ensure safe implementation in routine practice.

Nanotechnology holds significant potential to advance precision oncology in ER-positive breast cancer, as targeted nanoparticle drug delivery can enhance the therapeutic index by concentrating treatment within tumour cells while minimising toxicity to normal tissues [83]. When integrated with molecular profiling and ctDNA monitoring, nanocarriers may enable adaptive therapy, facilitate combination drug delivery and help overcome resistance mechanisms driven by mutations such as *ESR1* or *PIK3CA* [83].

Continued therapeutic development is essential as new treatments modify the natural history of ER-positive breast cancer and reveal novel resistance mechanisms. Most trials target ERs, cell cycle machinery and the *PIK3CA/AKT* pathway—often combined with an endocrine therapy backbone—highlighting the need for more effective endocrine agents. As novel combination therapies move into earlier treatment lines, newer treatment options are clearly needed upon progression. Since NGS-guided approaches focus on mutations, mutation-agnostic therapies like emerging ADCs and epigenetic modulators such as KAT2 inhibitors [84] remain important for patients without targetable mutations.

## 6. Conclusions

Molecular profiling is now a cornerstone to managing ER-positive advanced breast cancer, serving as a predictive and prognostic biomarker. International guidelines recommend routine implementation, using tissue or ctDNA, to identify actionable genomic alterations such as *PIK3CA* and *ESR1*, which has catalysed a surge in drug development and clinical trials, expanding treatment options for patients with advanced, incurable disease. Beyond treatment selection, molecular profiling may aid prognostication and monitoring of treatment response; however, critical unanswered questions regarding clinical validity, optimal integration into treatment pathways and impact on survival outcomes remain uncertain. Addressing real-world barriers is essential to ensure equitable access and facilitate routine implementation into clinical practice globally.

## Figures and Tables

**Table 1:** Relevant molecular alterations in ER-positive breast cancer classified as tier I or II according to the ESMO ESCAT scale [26]. Tier I identifies therapies suitable for routine clinical practice based on improved outcomes identified in clinical trials (IA randomised, IB prospective or IC basket trials). Tier II includes investigational therapies likely to provide clinical benefit based on either IIA prospective trials without survival endpoints or IIB retrospective studies [26].

Molecular Aberration	Estimated Prevalence in ER-Positive Breast Cancer	ESCAT Scale Classification	Drug Class Matched	Key Clinical Trial
*PIK3CA^mut^*	30–40% [17]	IA	PIK3CA inhibitors AKT1 inhibitors	SOLAR-1 [18] BYLieve [19] CAPItello-291 + 292 [21,27] INAVO120 [20]
*PTEN^del^*	7% [17]	I/II		CAPItello-291 + 292 [21,27]
*AKT1^mut^*	7% [17]	I/II		CAPItello-291 + 292 [21,27]
*ESR1^mut^*	Primary breast cancer: < 1% [15] Post adjuvant AI: 5% [16,17] Progression on AI for metastatic disease: 30–40% [16,17]	IA	SERDs	EMERALD [28] SERENA-6 [29]
Germline *BRCA1/2^mut^*	4% [15]	IA	PARP inhibitors	OlympiAD [30] EMBRACA [31]
Somatic *BRCA1/2^mut^*	3% [15]	IIB		TBCRC-048 [32]
*HER2^mut^*	3–4% [15]	IIB	Pan-HER TKIs Anti-HER2 ADCs	SUMMIT [33] DESTINY-PanTumor01 (DPT-01) [34]
*NTRK*fusions	<1% [26]	IC	NTRK inhibitors	LOXO-TRK-15002 [35]
Germline *PALB2^mut^*	1% [26]	IIB	PARP inhibitors	TBCRC-048 [32]

Abbreviations: ADC: Antibody–drug conjugate; AKT1: Protein kinase B; BRCA1/2: Breast cancer gene 1 and 2; ESCAT: ESMO Scale for Clinical Actionability of Molecular Targets; ESR1: Oestrogen receptor 1; HER2: human epidermal growth factor 2; NTRK: Neurotrophic tyrosine receptor kinase; PALB2: partner and localiser of BRCA2; PARP: Poly (ADP-ribose) polymerase; PIK3CA: phosphatidylinositol-4,5-bisphosphonate 3-kinase catalytic subunit alpha; PTEN: Phosphatase and tensin homolog; SERD: Selective oestrogen receptor degrader; TKI: Tyrosine kinase inhibitor.

**Table 2:** *ESR1^mut^* subtypes, their prevalence and clinical implications in ER-positive breast cancer. This table summarises key ESR1 ligand-binding domain mutations, their frequency in primary, recurrent and advanced disease and associated clinical outcomes. It highlights differences in prognosis and endocrine resistance among specific mutations, providing an understanding of how individual *ESR1^mut^* influence disease course.

*ESR1*	Prevalence	Median OS (Months) [38]
*ESR1^wt^*	NA	32.1 (95% CI 28.1–36.4)
*ESR1^mut^* (all)	Primary breast cancer: <1% [15] Post adjuvant AI: 5% [16,17] Progression on AI for metastatic disease: 30–40% [16,17]	20.7 (HR 1.6, 95% CI 17.7–28.1, *p* < 0.001)
*ESR1^mut^ D538G*	21.1% [38]–41.2% [39]	26.0 (HR 1.4, 95% CI 19.2–32.4, *p* = 0.03)
*ESR1^mut^ Y537S*	13.3% [38]–22.1% [39]	20.0 (HR 1.8, 95% CI 13.0–29.3, *p* = 0.003)
Dual mutations	3.8% [39]–5.5% [38]	15.2 (HR 2.23, 95% CI 10.9–27.4, *p* < 0.001)

Abbreviations: AI: aromatase inhibitor; HR: Hazard ratio; OS: Overall Survival; NA: Not applicable.

**Table 3:** Summary of phase II and III clinical trials investigating novel endocrine therapies in ER-positive/HER2-negative advanced breast cancer, stratified by ESR1 mutation status. * Negative trial.

Trial	Novel Drug and Mechanism	Phase	Line	Population	Treatment Arms	Prior CDK4/6i (%)	*ESR1^mut^* (%)	Efficacy *ESR1 ^mut^*	Efficacy *ESR1^wt^*	Efficacy Overall Population
EMERALD [28]	Elacestrant (PO SERD)	3	2–3	ER+/HER2− MBC, post-ET+ CDK4/6i	Elacestrant vs. PCET	100	48	PFS 3.8 vs. 1.9 mo; HR 0.55, 95% CI 0.39–0.77, *p* = 0.0005	NR	PFS 2.8 vs. 1.9 mo; HR 0.70, 95% CI 0.55–0.88, *p* = 0.002
SERENA-2 [60]	Camizestrant (PO SERD + ER antagonist)	2	2	ER+/HER2− MBC, post-ET	Camizestrant (75 mg, 100 mg, and 300 mg) vs. Fulvestrant	51	38	PFS 6.3 (75 mg) vs. 2.2 mo; HR 0.33, 90% CI 0.18–0.58	PFS 7.2 (75 mg) vs. 7.2 mo; HR 0.80, 90% CI 0.51–1.27	PFS 7.2 (75 mg) vs. 3.7 mo; HR 0.59, 90% CI 0.42–0.82, *p* = 0.017
EMBER-3 [61]	Imlunestrant (PO SERD)	3	1–2	ER+/HER2− MBC, post-ET ± CDK4/6i	Imlunestrant vs. Imlunestrant + Abemaciclib vs. PCET	58	38	Imlunestrant vs. PCET: PFS 5.5 vs. 3.8 mo; HR 0.76, *p* <0.001 Imlunestrant + Abemaciclib vs. Imlunestrant: PFS 9.4 vs. 5.5 mo; HR 0.57, 95% CI 0.44–0.73, *p* < 0.001	NR	Imlunestrant vs. PCET: PFS 5.6 vs. 5.5 mo; HR 0.87, 95% CI 0.72–1.04, *p* = 0.12
* AMEERA-3 [64]	Amcenestrant (PO SERD)	2	2–3	ER+/HER2− MBC, post-ET	Amcenestrant vs. PCET	79	44	PFS 3.7 vs. 2.0, HR 0.9, 95% CI 0.57–1.5	PFS 3.5 vs. 3.9 mo; HR 1.3, 95% CI 0.88–1.9	PFS 3.6 vs. 3.7 mo; HR 1.05 (95% CI: 0.79–1.40), *p* = 0.64
* AMEERA-5 [65]	Amcenestrant (PO SERD)	3	1	ER+/HER2− MBC without prior therapy	Amcenestrant + Palbociclib vs. Letrozole + Palbociclib	0	NR	NR	NR	Stopped for futility; mPFS estimates not robust. HR 1.2, 95% CI 0.93–1.55, *p* = 0.93
acelERA [62]	Giredestrant (PO SERD)	2	2–3	ER+/HER2− MBC, post-ET ± CDK4/6i	Giredestrant vs. PCET	42	38	PFS 5.3 vs. 3.5 mo; HR 0.60, 95% CI 0.35–1.03	PFS 7.2 vs. 6.6 months, HR 1.01, 95% CI 0.64–1.60	PFS 5.6 vs. 5.4 mo; HR 0.81, 95% 0.60–1.1, *p* = 0.17
VERITAC-2 [63]	Vepdegestrant (PROTAC ER degrader)	3	2–3	ER+/HER2− MBC, post-ET ± CDK4/6i	Vepdegestrant vs. Fulvestrant	100	43	PFS 5.0 vs. 2.1 mo; HR 0.58, 955 CI 0.43–0.78, *p* < 0.001	NR	PFS 3.8 vs. 3.6 mo; HR 0.83, 95% CI 0.69–1.01), *p* = 0.07
ELAINE-1 [66]	Lasofoxifene (SERM)	2	2	ER+/HER2− MBC, post-ET+ CDK4/6i, *ESR1^mut^*	Lasofoxifene vs. Fulvestrant	100	100	PFS 5.5 vs. 3.7 mo; HR 0.69, 95% CI 0.43–1.1, *p* = 0.138	NA	PFS 5.5 vs. 3.7 mo; HR 0.69, 95% CI 0.43–1.1, *p* = 0.138
ELAINE-2 [67]	Lasofoxifene (SERM)	2	2–3	ER+/HER2− MBC, post-ET ± CDK4/6i, *ESR1^mut^*	Lasofoxifene + Abemaciclib (single arm)	96	100	ORR 55.6% (95% CI 33.7–75.4)	NA	ORR 55.6% (95% CI 33.7–75.4)
PADA-1 [68]	Fulvestrant (IM SERD)	3	1.5 (rising *ESR1^mut^* on 1L)	ER+/HER2− MBC on ET + Palbociclib	Continue ET + Palbociclib vs. Fulvestrant + Palbociclib	100	100	PFS 11.9 vs. 5.7 mo; HR 0.61, 95% CI 0.43–0.86, *p* = 0.004	NA	PFS 11.9 vs. 5.7 mo; HR 0.61, 95% CI 0.43–0.86, *p* = 0.0040
SERENA-6 [29]	Camizestrant (PO SERD + ER antagonist)	3	1.5 (rising *ESR1^mut^* on 1L)	ER+/HER2− MBC on ET + CDK4/6i	Continue ET + CDK4/6i vs. Camizestrant + CDK4/6i	100	100	PFS 16 vs. 9.2 mo; HR 0.44, 95% CI 0.31–0.60, *p* < 0.00001	NA	PFS 16 vs. 9.2 mo; HR 0.44, 95% CI 0.31–0.60, *p* < 0.00001
SERENA-4 [69]	Camizestrant (PO SERD + ER antagonist)	3	1	ER+/HER2− MBC without prior therapy	Camizestrant + Palbociclib vs. Anastrazole + Palbociclib	0	NR	Results awaited		
persevERA [70]	Giredestrant (PO SERD)	3	1	ER+/HER2− MBC without prior therapy	Giredestrant + Palbociclib vs. Letrozole + Palbociclib	0	NR	Results awaited		
ELAINE-3 [71]	Lasofoxifene (SERM)	3	2–3	ER+/HER2− MBC, post-ET ± CDK4/6i (Ribociclib or Palbociclib), *ESR1^mut^*	Lasofoxifene + Abemaciclib vs. Fulvestrant + Abemaciclib	100	100	Results awaited		

Abbreviations: 95% CI: 95% confidence interval; CDK4/6i: cyclin-dependent kinase 4/6 inhibitor; ER+: oestrogen receptor positive; ESR1: oestrogen receptor 1; ET: endocrine therapy; HER2: human epidermal growth factor 2; HR: Hazard ratio; MBC: metastatic breast cancer; mo: months; NA: Not applicable; NR: Not reported; PCET: physician-choice endocrine therapy; PFS: progression-free survival; PO: per oral; PROTAC: proteolysis targeting chimera; SERM: selective oestrogen receptor modulator; SERD: selective oestrogen receptor degrader.

**Table 4:** Summary of phase II and III clinical trials investigating PIK3CA/AKT1/PTEN pathway inhibitors in ER-positive/HER2-negative advanced breast cancer. * Negative trial.

Trial	Novel Drug and Mechanism	Phase	Line	Population	Treatment Arms	Prior CDK4/6i (%)	Mutant Population (%)	Detection Method for Genomic Profiling	Efficacy in Mutant Cohort	Efficacy in Non-Mutant Cohort	Efficacy Overall Population
SOLAR-1 [18]	Alpelisib (PIK3CA inhibitor)	3	2	ER+/HER2− MBC, post-ET	Alpelisib + Fulvestrant vs. placebo + Fulvestrant	6	29 (*PIK3CA*)	Tissue	PFS 11.0 vs. 5.7 mo; HR 0.65, 95% CI 0.50–0.85, *p* < 0.001	PFS 7.4 vs. 5.6 mo; HR 0.85, 95% CI 0.58–1.25	NR
BYLieve [19]	Alpelisib (PIK3CA inhibitor)	2–single arm	2	ER+/HER2− MBC, post-ET + CDK4/6i	Alpelisib + Fulvestrant	100	100 (*PIK3CA*)	Tissue or ctDNA	PFS 8.0 mo (95% CI 5.6–8.6)	NA	PFS 8.0 mo (95% CI 5.6–8.6)
SANDPIPER [77]	Taselisib (PIK3CA inhibitor)	3	2	ER+ MBC, post-ET	Taselisib + Fulvestrant vs. placebo + Fulvestrant	3	100 (*PIK3CA*)	Tissue	PFS 7.4 vs. 5.4 mo; HR 0.70, 95% CI 0.56–0.89, *p* = 0.0037	NA	PFS 7.4 vs. 5.4 mo; HR 0.70, 95% CI 0.56–0.89, *p* = 0.0037
* FERGI [78]	Pictilisib (PIK3CA inhibitor)	2	2	ER+/HER2− MBC, post-ET	Pictilisib + Fulvestrant vs. Fulvestrant + placebo	U	41 (*PIK3CA*)	Tissue	PFS 6.5 vs. 5.1 mo; HR 0.73, 95% CI 0.42–1.28, *p* = 0.268	PFS 5.8 vs. 3.6 mo; HR 0.72, 95% CI 0.42–1.23, *p* = 0.23	PFS 6.6 vs. 5.1 mo; HR 0.74, 95% CI 0.52–1.06, *p* = 0.096
CAPItello-291 [21]	Capivasertib (Pan-AKT inhibitor)	3	2	ER+ /HER2− MBC, post-ET± CDK4/6i	Capivasertib + Fulvestrant vs. Fulvestrant + placebo	70	41 (*PIK3CA*, *AKT1* or *PTEN*)	Tissue	PFS 7.3 vs. 3.1 mo; HR 0.50, 95% CI 0.38–0.65, *p* < 0.001	PFS 7.2 vs. 3.7 mo; HR 0.70, 95% CI 0.56–0.88 (post-hoc)	PFS 7.2 vs. 3.6 mo; HR 0.60, 95% CI 0.51–0.71, *p* <0.001
FINER [76]	Ipatasertib (Pan-AKT inhibitor)	3	2	ER+/HER2− MBC, post-ET+ CDK4/6i	Ipatasertib + Fulvestrant vs. Fulvestrant + placebo	100	44 (*PIK3CA*, *AKT1* or *PTEN*)	ctDNA	PFS 5.45 vs. 1.91 mo; HR 0.47, 95% CI 0.31–0.72, *p* = 0.0005	NR	PFS 5.32 vs. 1.94 mo; HR 0.61, 95% CI 0.46–0.81, *p* = 0.0007
INAVO120 [20]	Inavolisib (PIK3CA inhibitor)	3	1	ER+/HER2− MBC, PIK3CA-mutant, relapsed during or within 12 months of adjuvant ET	Inavolisib + Fulvestrant + Palbociclib vs. placebo + Fulvestrant + Palbociclib	1	100 (*PIK3CA*)	Tissue or ctDNA	PFS 15.0 vs. 7.3 mo; HR 0.43, 95% CI 0.32–0.59, *p* < 0.001	NA	PFS 15.0 vs. 7.3 mo; HR 0.43, 95% CI 0.32–0.59, *p* < 0.001
CAPItello-292 [27]	Capivasertib (Pan-AKT inhibitor)	3	1	ER+/HER2− MBC, relapsed during or within 12 months of adjuvant ET	Capivasertib + Fulvestrant + CDK4/6i (Ribociclib or palbociclib) vs. Fulvestrant + CDK4/6i (Ribociclib or palbociclib)	Results awaited					

Abbreviations: 95% CI: 95% confidence interval; AKT1: Protein kinase B; CDK4/6i: cyclin-dependent kinase 4/6 inhibitor; ctDNA: circulating tumour DNA; ER +: oestrogen receptor positive; ET: endocrine therapy; HER2: human epidermal growth factor 2; HR: Hazard ratio; MBC: metastatic breast cancer; mo: months; NA: Not applicable; NR: not reported; PFS: progression-free survival; PIK3CA: phosphatidylinositol-4,5-bisphosphonate 3-kinase catalytic subunit alpha.

## Abbreviations

The following abbreviations are used in this manuscript:

95% CI	95% confidence interval
ADC	Antibody–drug conjugate
AIs	Aromatase inhibitors
*AKT1*	Protein kinase B
ASCO	American Society of Clinical Oncology
*BRCA1/2*	Breast cancer gene 1 and 2
CDK4/6	Cyclin-dependent kinase 4/6
cfDNA	Cell-free DNA
ChT	Chemotherapy
ctDNA	Circulating tumour DNA
Dato-DXd	Datopotamab deruxtecan
ddPCR	digital droplet polymerase chain reaction
E2	Oestradiol
ER	Oestrogen receptor
ERα	Oestrogen receptor alpha
ERβ	Oestrogen receptor beta
ESCAT	ESMO Scale for Clinical Actionability of Molecular Targets
ESMO	European Society for Medical Oncology
*ESR1*	Oestrogen receptor 1
ET	Endocrine therapy
FDA	Food and Drug Administration
Fulv	Fulvestrant
GNRH	Gonadotropin releasing hormone
HER2	Human epidermal growth factor 2
HR	Hazard ratio
IHC	Immunohistochemistry
MBC	Metastatic breast cancer
Mo	Months
*MTOR*	Mammalian target of rapamycin
NA	Not applicable
NGS	Next-generation sequencing
NR	Not reported
*NTRK*	Neurotrophic tyrosine receptor kinase
OS	Overall survival
*PALB2*	Partner and localiser of BRCA2
PARP	Poly (ADP-ribose) polymerase
PDK1	3-phosphoinositide-dependant kinase 1
*PIK3CA*	phosphatidylinositol-4,5-bisphosphonate 3-kinase catalytic subunit alpha
PIP2	Phosphatidylinositol (4,5)-bisphosphonate
PIP3	Phosphatidylinositol (3,4,5)-Triphosphonate
PFS	Progression-free survival
PR	Progesterone receptor
*PTEN*	Phosphatase and tensin homolog
*Rb*	Retinoblastoma
SERD	Selective oestrogen receptor degrader
SERM	Selective oestrogen receptor modulator
SG	Sacituzumab Govitecan
T	Testosterone
T-DXd	Trastuzumab Deruxtecan
TFI	Treatment-free interval
TKI	Tyrosine kinase inhibitor
